# Influence of variable stiffness shoes in sports performance and protection of lower extremity injury

**DOI:** 10.1186/1757-1146-5-S1-O50

**Published:** 2012-04-10

**Authors:** Jee-Chin Teoh, Wen-Min Chen, Taeyong Lee

**Affiliations:** 1 Division of Bioengineering, National University of Singapore, Singapore

## Background

Footwear is an effective biomechanical solution to lower extremity joint problems. Variable stiffness shoe (VSS) is designed. It has been proved to reduce knee internal abduction (external adduction) moment [1]. This helps to slow down progression of medial knee osteoarthritis (OA) [2]. However, there is no study on the effects of VSS on lower extremity during dynamic activities besides walking. Influence of VSS in sports performance is also yet to be examined. This study aims to investigate the biomechanical influence of VSS on lower extremity during dynamic activities and to assess the potential of VSS in improving sports performance.

## Materials and methods

**Figure 1:**
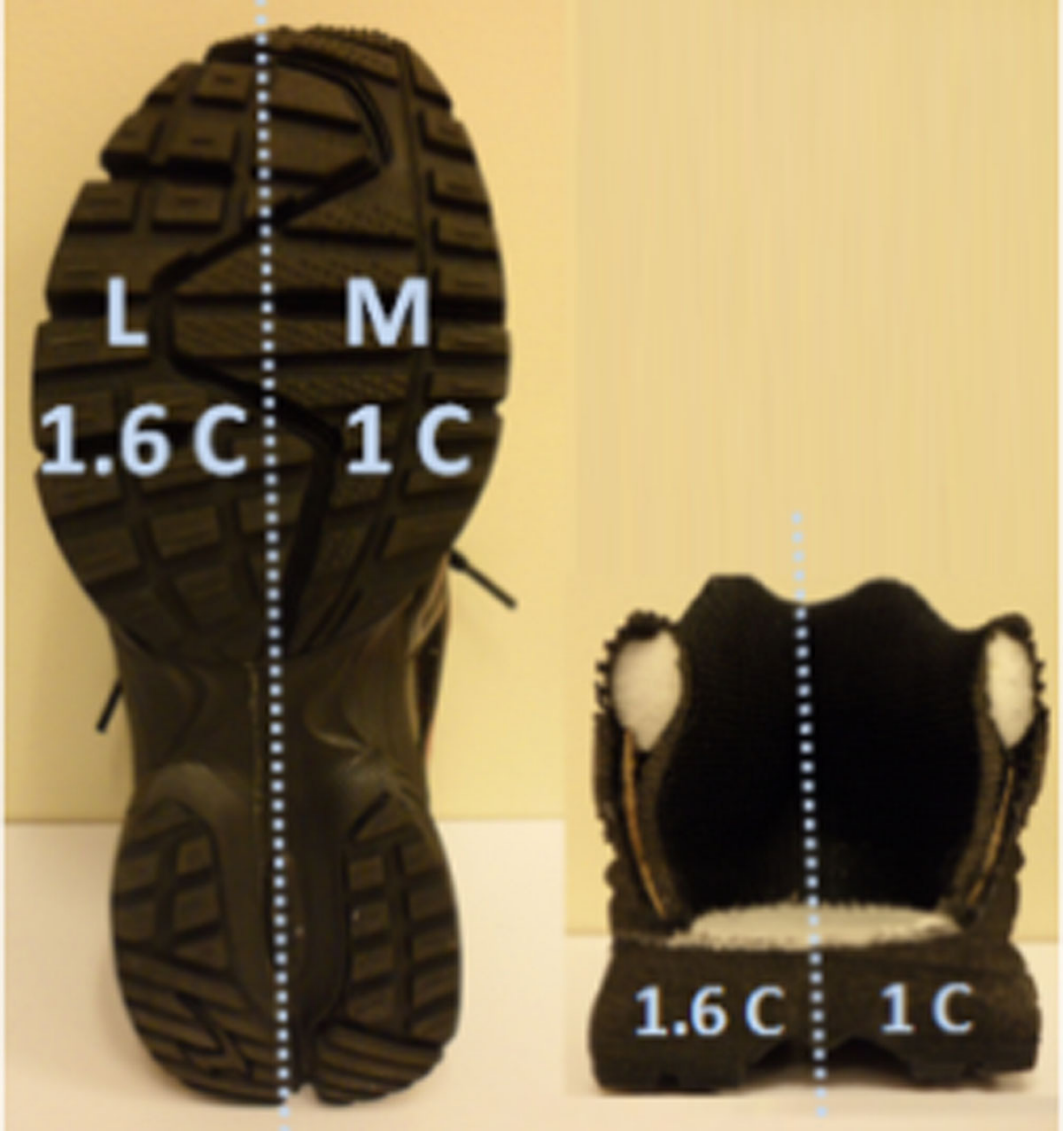
Images of shoe bottom and shoe cross section showing lateral (L) and media (M) soles.

15 female and 15 male subjects walked in 2 conditions: VSS and Control. VSS had a lateral sole 1.6 times stiffer than medial (Figure 1). The optimized ratio was obtained from finite element analysis of a simplified 2D knee model. Control had uniform stiffness outsole.3D kinematic and kinetic analysis was conducted during walking, running, stop jumping and lateral hopping. Rating on footwear comfort was also performed.

## Results

Increased posterior force during running and stop jumping (Table 1) ensured controlled gait termination and reduced the risk of fall. Increased anterior force during walking and running (Table 1) increased forward propulsion and acceleration. Knee internal abduction moment was generally reduced (Table 1). This showed potential of VSS as sportswear that helped to relieve medial knee loading in more vigorous activities such as running, stopping and jumping. Kinetic data and comfort data showed that VSS did not change gait kinematics much. Rating differences were all insignificant (p<0.05). The stiffness variation in VSS was hardly noticeable. Shoe comfort was not compensated in VSS.

**Table 1:** Table compares only the averages of kinematics (angles) and kinetics (moments and forces) data that are statistically significant (p<0.05) during the dynamic activities.

Activity	Joint	Variable Name	Control	VSS	%difference
Walking	Knee	Max adduction moment (%BWxHt)	0.37	0.35	-5.994
Walking		Max anterior force at push off (%BW)	19.73	20.97	6.302
Running	Knee	Max adduction moment (%BWxHt)	1.04	0.88	-14.716
Running		Max posterior force (%BW)	-24.50	-28.93	18.072
Running		Max anterior force (%BW)	29.02	30.78	6.078
Stop Jumping	Knee	Max adduction moment (%BWxHt)	0.83	0.67	-18.661
Stop Jumping		Max posterior force (%BW)	-67.67	-73.33	8.373

## Conclusion

The study demonstrated great potential of VSS in improving sports achievement and protecting knee. Outsole configuration can be further modified by varying outsole stiffness along anteroposterior axis for better performance and protection.
